# Patterns of Anatomic Injury in Critically Injured Combat Casualties: A Network Analysis

**DOI:** 10.1038/s41598-019-50272-3

**Published:** 2019-09-24

**Authors:** Jud C. Janak, Edward L. Mazuchowski, Russ S. Kotwal, Zsolt T. Stockinger, Jeffrey T. Howard, Frank K. Butler, Jonathan A. Sosnov, Jennifer M. Gurney, Stacy A. Shackelford

**Affiliations:** 1 Department of Defense Joint Trauma System, Defense Health Agency, Joint Base San Antonio–Fort Sam Houston, Texas, USA; 2 Armed Forces Medical Examiner System, Dover Air Force Base, Dover, Delaware USA; 3 Department of Military and Emergency Medicine, Uniformed Services University of the Health Sciences, Bethesda, Maryland USA; 4 Department of Pathology, Uniformed Services University of the Health Sciences, Bethesda, Maryland USA; 5 Texas A&M Health Science Center College of Medicine, College Station, USA; 6 Navy Medicine Readiness and Training Command Jacksonville, Jacksonville, FL USA; 7 Department of Kinesiology, Health, and Nutrition, The University of Texas at San Antonio, San Antonio, USA; 8 375th Medical Group, Scott Air Force Base, Illinois, USA

**Keywords:** Epidemiology, Medical research

## Abstract

A mortality review of death caused by injury requires a determination of injury survivability prior to a determination of death preventability. If injuries are nonsurvivable, only non-medical primary prevention strategies have potential to prevent the death. Therefore, objective measures are needed to empirically inform injury survivability from complex anatomic patterns of injury. As a component of injury mortality reviews, network structures show promise to objectively elucidate survivability from complex anatomic patterns of injury resulting from explosive and firearm mechanisms. In this network analysis of 5,703 critically injured combat casualties, patterns of injury among fatalities from explosive mechanisms were associated with both a higher number and severity of anatomic injuries to regions such as the extremities, abdomen, and thorax. Patterns of injuries from a firearm were more isolated to individual body regions with fatal patterns involving more severe injuries to the head and thorax. Each injury generates a specific level of risk as part of an overall anatomic pattern to inform injury survivability not always captured by traditional trauma scoring systems. Network models have potential to further elucidate differences between potentially survivable and nonsurvivable anatomic patterns of injury as part of the mortality review process relevant to improving both the military and civilian trauma care systems.

## Introduction

Injury is the leading cause of death for US adults under the age of 45^1^. Approximately 20% of these trauma deaths are considered potentially preventable^2,3^. To address the public health burden from these preventable deaths, the National Academies of Sciences, Engineering, and Medicine recently published a report with recommendations to mitigate death from trauma^2^. These recommendations included integrating lessons learned from recent military conflicts into a national learning trauma care system to achieve zero preventable deaths. Data from injured service members can help improve the national trauma care system by providing a better understanding of injury survivability^4^, and identifying medical and non-medical interventions that ultimately reduce preventable deaths.

For a trauma death to be preventable, the underlying injury burden must first be evaluated and determined to be considered survivable or potentially survivable^5^. Paramount is determining which injury or constellation of injuries consistently fit criteria for survivability. Determination of injury survivability has traditionally been based on subject matter expert opinion, physiologic injury severity, and/or anatomic injury severity^6^. However, a requirement for a better understanding of the association between survivability and whole-body patterns of anatomic injuries persists as traditional methods (1) do not fully account for the complexity of explosive and firearm polytraumatic injuries; (2) are overly reliant on an injury severity score (ISS) initially designed primarily for civilian motor vehicle crashes; and (3) inadvertently minimize the extent of damage by concentrating on isolated rather than whole-body patterns of anatomic injuries. To address these issues, and to gain a better understanding of survivability among fatalities and survivors, we investigated whole-body patterns of anatomic injuries among combat casualties using binary network analysis^7^.

## Results

### Description of fatal and non-fatal battle-related Injured US Service Members

Of 5,344 battle-related deaths identified by the Armed Forces Medical Examiner System (AFMES), a total of 4,320 fatalities met study inclusion criteria (eFig. 1). Of 83,839 casualties identified in the Department of Defense Trauma Registry (DODTR), a total of 1,383 met study inclusion criteria. There were no meaningful differences between fatalities and survivors regarding age, sex, or military service (Table 1). The study population had a higher proportion of fatalities versus survivors from Iraq combat operations (64.9% vs. 52.9%, p < 0.001). Overall, fatalities suffered more severe injuries with an ISS >50 (50% vs. 5%, p < 0.001). Except for critical injuries to the lower extremity, fatalities had a higher proportion of sustaining at least one of the highest injury severity group injuries for each anatomic region. Compared to survivors, over three times the proportion of fatalities sustained at least one of these highest severity group injuries to the head, face, neck, thorax, abdomen, spine, upper extremity, and/or lower extremity (42.4% vs. 13.5%, p < 0.001).

**Table 1:** Demographic and Injury Characteristics of US Military Casualties who Sustained Battle-Related Critical Injuries (Injury Severity Scale Score, 25–75) during Combat Operations in Iraq and Afghanistan (2001–2014), Fatalities versus Survivors.

	Total (n = 5,703)	Fatalities (n = 4,320)	Survivors (n = 1,383)	Test Statistic^a^ (p-value)
Injury Year-No. (%)				**118.8** **(<0.001)**
2001–2007	3,269 (57.3)	2,575 (59.6)	694 (50.2)	
2008–2014	2,262 (39.7)	1,573 (36.4)	689 (49.8)	
Missing	172 (3.0)	172 (4.0)	0 (0.0)	
Age-No. (%)				**4.6** **(0.21)**
18–21 years	1,561 (27.4)	1,162 (26.9)	399 (28.9)	
22–29 years	2,883 (50.6)	2,182 (50.5)	701 (50.7)	
30–35 years	715 (12.5)	547 (12.7)	168 (12.2)	
>35 years	544 (9.5)	429 (9.9)	115 (8.3)	
Sex-No. (%)				**2.2** **(0.14)**
Male	5622 (98.6)	4,253 (98.5)	1,369 (99.0)	
Female	81 (1.4)	67 (1.6)	14 (1.0)	
Service-No. (%)				**1.5** **(0.68)**
Army	4,154 (72.8)	3,160 (73.2)	994 (71.9)	
Marine Corps	1,306 (22.9)	975 (22.6)	331 (23.9)	
Navy	159 (2.8)	119 (2.8)	40 (2.9)	
Air Force	84 (1.5)	66 (1.5)	18 (1.3)	
Primary Country (Combat Operation)-No. (%)				**63.6** **(<0.001)**
Iraq (OIF^b^/OND^c^)	3,535 (62.0)	2,803 (64.9)	732 (52.9)	
Afghanistan (OEF^d^)	2,168 (38.0)	1,517 (35.1)	651 (47.1)	
Injury Mechanism-No. (%)				**75.0** **(<0.001)**
Explosive	4,106 (72.0)	3,037 (70.3)	1,069 (77.3)	
Firearm	1,289 (22.6)	988 (22.9)	301 (21.8)	
Non-Explosive, Non-Firearm	308 (5.4)	295 (6.8)	13 (0.94)	
Injury Severity Score-No. (%)				**876.8** **(<0.001)**
25–50	3,493 (61.3)	2,179 (50.4)	1,314 (95.0)	
51–75	2,210 (38.8)	2,141 (49.6)	69 (5.0)	
Max AIS^e^ Severity-No. (%)				
Head Severity 6	650 (11.4)	643 (14.9)	7 (0.5)	**(<0.001)**
Face Severity 4	16 (0.3)	6 (0.1)	10 (0.7)	**(0.001)**
Neck Severity 6	95 (1.7)	95 (2.2)	0 (0.0)	**(<0.001)**
Thorax Severity 6	521 (9.1)	514 (11.9)	7 (0.5)	**(<0.001)**
Abdomen Severity 6	404 (7.1)	404 (9.4)	0 (0.0)	**(<0.001)**
Spine Severity 6	247 (4.3)	234 (5.4)	13 (0.9)	**(<0.001)**
Upper Extremity Severity 5	57 (1.0)	54 (1.3)	3 (0.2)	**(<0.001)**
Lower Extremity Severity 5	604 (10.6)	457 (10.6)	147 (10.6)	**0.003** **(0.96)**
Total^f^	2,017 (35.4)	1,831 (42.4)	186 (13.5)	**383.7** **(<0.001)**

^a^Test Statistic: Chi-Square Test or Fisher’s Exact Test as appropriate; ^b^OIF: Operation Iraqi Freedom, primarily Iraq; ^c^OND: Operation New Dawn, primarily Iraq; ^d^OEF: Operation Enduring Freedom, primarily Afghanistan; ^e^AIS: Abbreviated Injury Scale-2005; ^f^The number of casualties that sustained one or injuries in the following anatomic severity groups: head injury severity 6, face injury severity 4, neck injury severity 6, thorax injury severity 6, abdomen injury severity 6, spine injury severity 6, upper extremity injury severity 5, and/or lower extremity injury severity 5.

### Network structures

**Figure 1:**
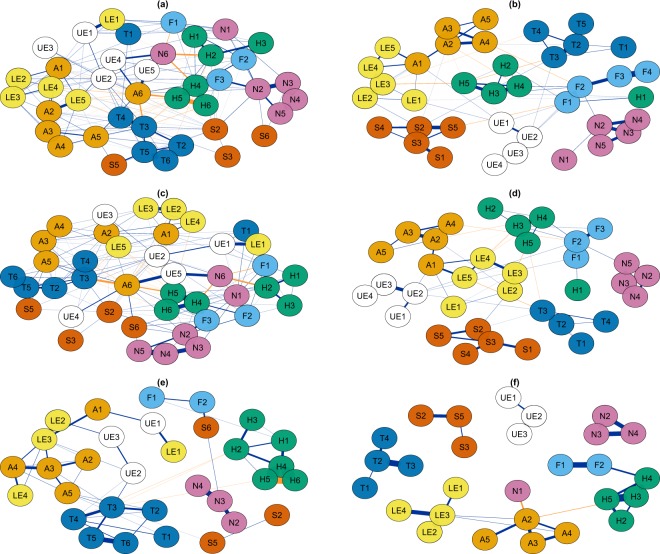
Network Structures of Anatomic Body Regions among US Military Casualties who Sustained Battle-Related Critical Injuries (Injury Severity Scale Score, 25–75): (**a**) All Fatalities, (**b**) All Survivors, (**c**) Explosive Mechanism Fatalities, (**d**) Explosive Mechanism Survivors, (**e**) Firearm Mechanism Fatalities, (**f**) Firearm Mechanism Survivors. Individual injuries grouped by both the anatomic body region injured (H: Head (green nodes), F: Face (light blue nodes), N: Neck (purple nodes), T: Thorax (dark blue nodes), A: Abdomen (orange nodes), S: Spine (dark orange nodes), UE: Upper Extremity (white nodes), LE: Lower Extremity (yellow nodes)) and severity of the injury (1: minor, 2: moderate, 3: serious, 4: severe, 5: critical, 6: maximum). For example, A3 is equivalent to all Abbreviated Injury Scale-2005 Injuries to the abdomen with a severity of 3 or serious. Navy edge colors represent positive associations and orange edge colors represent negative associations.

Reported in Fig. 1 are overall and injury mechanism specific empirical network structures. This resulted in empirical graphs of 28 (Fig. 1f), 32 (Fig. 1e), 35 (Fig. 1d), 38 (Fig. 1b), and 41(Fig. 1a,c) anatomic injury groups that met inclusion criteria resulting in 378, 496, 595, 703, and 820 potential connections, respectively. The total number of within and between body region associations was higher for all three fatal network model populations: total (180 vs. 75), explosive mechanism (169 vs. 61), and firearm mechanism (52 vs. 29) (Table 2). All networks were considered borderline smallworld, as they had a high global clustering coefficient and short average shortest path with a smallworldness between 1 and 3^8,9^. In general, compared to survivors, fatalities had a higher number and stronger connections among anatomic body regions with higher injury severity (eFig. 2). In addition, more severe injuries to the thorax and abdomen showed higher betweenness (i.e. connections with other anatomic injury groups) and closeness (i.e. clustering with other anatomic injury groups).

**Table 2:** Description of Total Population and Subgroup Injury Mechanism Population Anatomic Network Models for US Military Casualties who Sustained Critical Injuries (Injury Severity Scale Score, 25–75), Fatalities vs. Survivors.

	Total		Explosive Mechanism		Firearm Mechanism	
	Fatalities	Survivors	Fatalities	Survivors	Fatalities	Survivors
Number of Edges	180	75	169	61	52	29
Number of Positive Edges	150	65	143	52	48	27
Number of Negative Edges	30	10	26	9	4	2
Average Shortest Path	2.01	3.05	2.10	3.20	4.06	19.9
Transitivity	0.41	0.30	0.42	0.28	0.32	0.38
Smallworldness	1.25	2.14	1.39	2.36	2.06	2.48

Fatal anatomic injuries caused by explosive mechanisms clustered into multiple body regions (Fig. 1c). For example, more severe lower extremity injures (LE5, yellow) cluster with abdominal injuries (A2-A5, orange), and injuries to the thorax (T3-T4, dark blue). Conversely, survivor anatomic injuries resulting from explosive mechanism tend to cluster into isolated body regions (Fig. 1d).

When examining injuries resulting from firearm mechanism (Fig. 1e,f), both fatal and survivor anatomic injuries tend to be isolated to individual body regions such as the head and neck regions. However, fatal injuries tend to cluster more closely around the following: (1) more severe head injuries (H4-H6, green); (2) more severe injuries to the thorax (T4-T6, dark blue); and (3) injuries to the lower extremities (LE2-LE4, yellow), abdomen (A1-A5, orange), and upper extremities (UE2 and UE3, white).

### Within-anatomic body region associations

**Figure 2:**
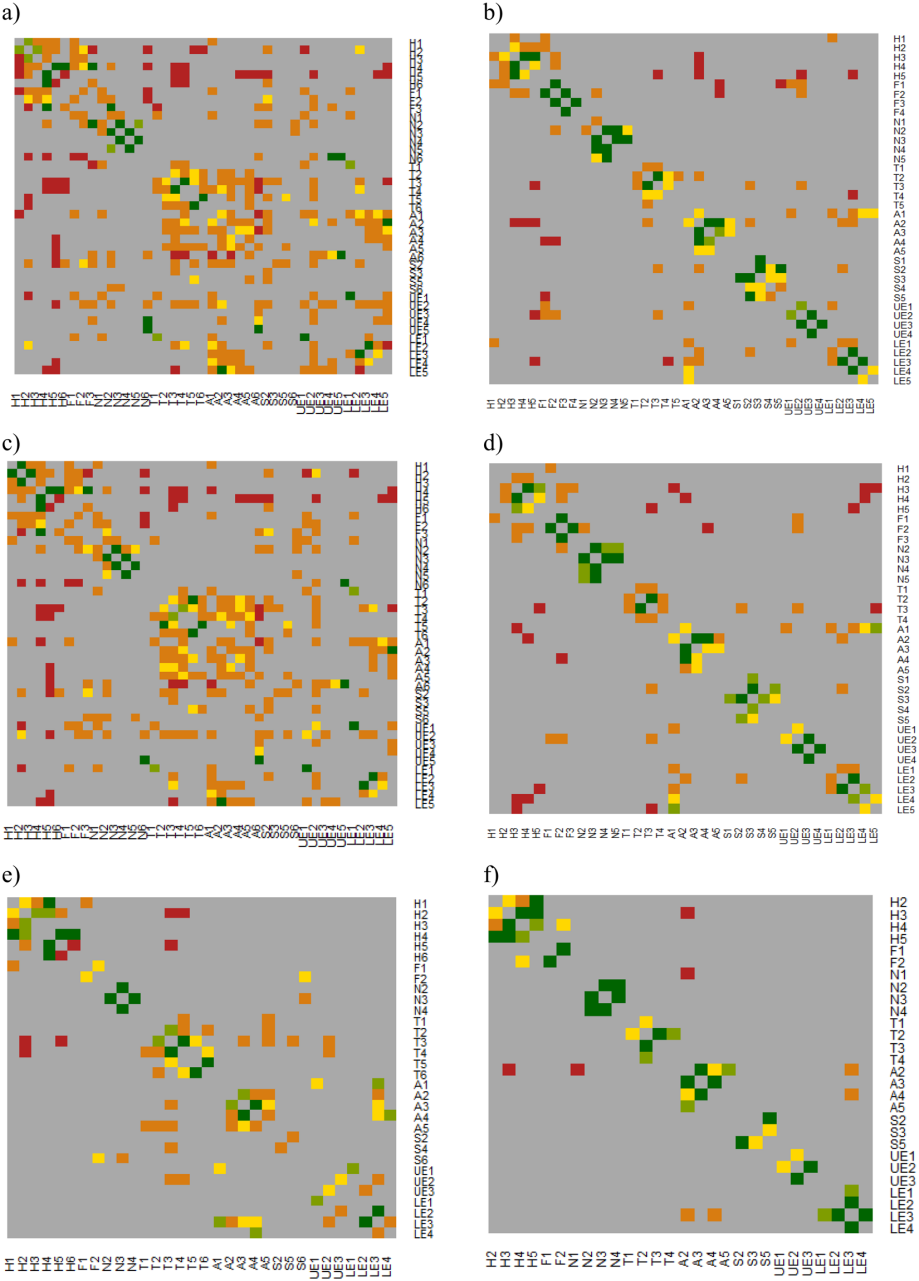
Heat Map of Associations Within and Between Anatomic Body Regions among US Military Casualties who Sustained Battle-Related Critical Injuries (Injury Severity Scale Score, 25–75): (**a**) All Fatalities, (**b**) All Survivors, (**c**) Explosive Mechanism Fatalities, (**d**) Explosive Mechanism Survivors, (**e**) Firearm Mechanism Fatalities, (**f**) Firearm Mechanism Survivors. Individual injuries are grouped by anatomic body region (H: Head, F: Face, N: Neck, T: Thorax, A: Abdomen, S: Spine, UE: Upper Extremity, LE: Lower Extremity) and severity (1: minor, 2: moderate, 3: serious, 4: severe, 5: critical, 6: maximum). For example, A3 is equivalent to all Abbreviated Injury Scale-2005 Injuries to the abdomen with a severity of 3 or serious. Associations reported as odds ratios: Dark red: (OR: >0.00–1.00); Dark Orange: (OR: >1.00–2.00); Dark Yellow (OR: >2.00–3.00); Light Green (OR: >3.00–4.00); Dark Green (OR: >4.00).

The majority of the strongest positive associations for within-anatomic body regions were the same for both fatal and survivor injuries (Fig. 2, eTables 5 and 6). The main difference was that fatal positive associations involved more severe injuries. Among fatal injuries sustained by explosive mechanism (Fig. 2c, eTable 5), the strongest within-thorax region injury association was between severity 6 and severity 5 injuries (OR: 11.2), while for survivor injuries (Fig. 2d, eTable 6) it was between severity 3 and severity 2 injuries (OR: 5.5). For fatal injuries sustained by firearm mechanism (Fig. 2e, eTable 5), the two strongest within-head region injury associations were between severity 5 and severity 4 injuries (OR: 80.2) and severity 6 and severity 4 injuries (OR: 75.6). For survivor injuries from firearm mechanism (Fig. 2f, eTable 6), the two strongest within-head injury associations were between severity 5 and severity 3 injuries (OR: 25.3) and severity 4 and severity 3 injuries (OR: 10.7).

### Between-anatomic body region associations

The proportion of between-anatomic body region associations were notably higher for fatal injuries in the total population. Compared to survivor injuries, the proportion of between-anatomic region associations for the total population was more than two times higher for fatal injuries (Tables 3 and 4). This was particularly driven by fatal injuries from explosive mechanism. Also, the proportion of fatal between-anatomic body region associations were more than two times higher compared to survivors (eTables 1 and 2): head (16% vs. 7%), neck (10% vs. 1%), face (22% vs. 8%), spine (16% vs. 1%), thorax (19% vs. 3%), upper extremities (16% vs. 3%), abdomen (23% vs. 6%), and lower extremities (17% vs. 7%). Compared to injuries from an explosive mechanism, injuries from a firearm were more isolated to individual body regions such as the head and neck for both fatal and survivor injuries. However, compared to survivor injuries, fatal injuries from a firearm had a higher proportion of associations between other body regions (eTables 3 and 4): head (3% vs. 2%), face (3% vs. 2%), spine (3% vs. 0%), thorax (6% vs. 0%), upper extremities (7% vs. 0%), abdomen (7% vs. 4%), and lower extremities (6% vs. 2%).

**Table 3:** Number of Connections Within and Between Anatomic Injury Groups among Fatalities of US Military Casualties who Sustained Battle-Related Critical Injuries (Injury Severity Scale Score, 25–75) from all Mechanisms Combined (N = 4,320).

	Head	Neck	Face	Spine	Thorax	Upper Extremity	Abdomen	Lower Extremity
Head	**11/15 (73)**							
Neck	3/36 (8)	**6/15 (40)**						
Face	10/18 (56)	6/18 (33)	**1/3 (33)**					
Spine	3/24 (13)	3/24 (13)	2/12 (17)	**2/6 (33)**				
Thorax	8/36 (22)	1/36 (3)	1/18 (6)	4/24 (17)	**11/15 (73)**			
Upper Extremity	2/30 (7)	5/30 (17)	4/15 (27)	1/20 (5)	4/30 (13)	**3/10 (30)**		
Abdomen	4/36 (11)	3/36 (8)	2/18 (11)	4/24 (17)	16/36 (44)	8/30 (27)	**11/15 (73)**	
Lower Extremity	4/30 (13)	1/30 (3)	1/15 (7)	1/20 (5)	4/30 (13)	9/25 (36)	16/30 (53)	**5/10 (50)**
***All other Anatomic Regions***	***34/210 (16)***	***22/210 (10)***	***26/114 (23)***	***18/148 (12)***	***38/210 (18)***	***33/180 (18)***	***53/210 (25)***	***36/180 (20)***

Number of connections within and between Abbreviated Injury Scale-2005 anatomic injury groups relative to the maximum number of possible connections. Within anatomic region associations are in **bold**.

**Table 4:** Number of Connections Within and Between Anatomic Injury Groups among Survivors of US Military Casualties who Sustained Battle-Related Critical Injuries (Injury Severity Scale Score, 25–75) from all Mechanisms Combined (N = 1,383).

	Head	Neck	Face	Spine	Thorax	Upper Extremity	Abdomen	Lower Extremity
Head	**7/10 (70)**							
Neck	0/25 (0)	**6/10 (60)**						
Face	4/20 (20)	1/20 (5)	**3/6 (50)**					
Spine	0/25 (0)	0/25 (0)	1/20 (5)	**7/10 (70)**				
Thorax	1/25 (4)	0/25 (0)	0/20 (0)	1/25 (4)	**6/10 (60)**			
Upper Extremity	1/20 (5)	0/20 (0)	3/16 (19)	0/20 (0)	1/20 (5)	**3/6 (50)**		
Abdomen	3/25 (12)	0/25 (0)	2/20 (10)	1/25 (4)	1/25 (4)	1/20 (5)	**6/10 (60)**	
Lower Extremity	2/25 (8)	0/25 (0)	0/20 (0)	1/25 (4)	1/25 (4)	1/20 (5)	6/25 (24)	**5/10 (50)**
***All other Anatomic Regions***	***11/165 (7)***	***1/165 (1)***	***11/136 (8)***	***4/165 (2)***	***5/165 (3)***	***7/136 (5)***	***14/165 (8)***	***11/165 (7)***

Number of connections within and between Abbreviated Injury Scale-2005 anatomic injury groups relative to the maximum number of possible connections. Within anatomic region associations are in **bold**.

The vast majority of the top 10 positive associations for both fatal and survivor injuries were all within the same anatomic body region (Fig. 2, eTables 5 and 6). However, compared to survivor injuries, fatal injuries were often distinguished by positive associations with other body regions. For injuries from explosive mechanism (Fig. 2c,d), lower extremity injuries of severity 3 and 2 had a strong positive association for both fatal (OR: 5.5) and survivor injuries (OR: 7.2). However, fatal injuries with a lower extremity severity 3 were also positively associated with abdominal injuries of severities 1–4 (ORs: 1.3–1.4), thorax injuries of severity 3 (OR: 2.3), and upper extremity injuries of severity 2 (OR: 1.1). Survivor injuries had no positive associations with any of these other body regions. Similarly, for injuries from firearm mechanism (Fig. 2e,f), strong positive associations were reported for neck injuries of severity 4 with neck injuries of severity 3 for both fatal (OR: 12.6) and survivor injuries (OR: 8.7). However, fatal neck injuries of severity 3 were also associated with spine injuries of severity 6 (OR: 1.7) and survivor injuries were not associated with these maximum injuries to the spine.

## Discussion

Eliminating preventable death from injury requires a multifactorial approach. In addition to medical and non-medical considerations that provide an appropriate and overarching context for death preventability^10^, the foundation of this approach must also include a detailed understanding of injury survivability. Anatomic body regions contain innate structures, vessels, fluids, and organs that vary in injury sensitivity and durability, as well as overall importance for sustaining life. Each injury within each anatomic body region produces an individual burden and degree of severity that collectively contributes to an injury pattern, whole-body burden, and degree of survivability.

Our novel study and analytic approach investigated whole-body patterns of anatomic injuries and associated survivability. Key findings from our study included: (1) some injury patterns had no documented evidence of survival and therefore were most likely non-survivable; (2) fatal injuries were more likely to have higher severity of injuries within the same anatomic body region and also include multiple anatomic body regions; and (3) explosive injuries most often involved multiple anatomic body regions whereas firearm injuries were more often isolated within specific body regions.

Our analyses found several groups of anatomic injuries that either have no documented evidence of survival or that survival was exceedingly rare. No casualties survived with maximum injuries (i.e. AIS severity 6) to the neck or abdomen. Only 30 casualties, or approximately 2%, lived with one or more maximum injuries (AIS severity 6) to the head (n = 7), thorax (n = 7), or spine (n = 13), or critical injuries (AIS severity 5) to the upper extremity injuries (n = 3). It is also not the case that these injuries were less prevalent, as over one-third of fatalities (n = 1,515; 35%) sustained one or more injuries within these critical or maximum injury severity groups to the head, neck, spine, thorax, abdomen, and/or upper extremities. Further inspection of injuries within these body regions will highlight which specific injuries have no evidence of survival. Many of the injuries within these highest injury severity groups overlap with injuries considered non-survivable in previous publications based on subject matter expert opinion^11–14^, and thus lend empirical support for these opinions.

These results also show the importance of injury mechanism in understanding anatomic patterns of injuries within populations of fatalities and survivors. This should not be surprising given what is known about how different mechanisms of injury impact subsequent pathophysiologic derangement^15–17^. Explosive mechanisms can generate indiscriminate polytraumatic injuries with unique pathophysiologic characteristics resulting from each component of primary, secondary, tertiary, and quaternary blast effects^15^. On the other hand, firearm mechanisms have their own unique considerations as it relates to ballistic properties, impact pathophysiology, and subsequent tissue disruption resulting from different weapons systems and ammunition^16^. Injuries and pathophysiologic derangement from explosive mechanisms involve multiple body regions, while firearm injuries are more isolated to specific body regions, such as to the head and thorax as seen in our study population. These results are consistent with an analysis performed from the Israeli National Trauma Registry on over 1,000 terror-related injuries from explosive and firearm mechanisms^18^. Compared to injuries from a firearm mechanism, the Israeli analysis found that injuries from explosive mechanisms were more likely to involve three or more anatomic body regions and injuries categorized as critical (i.e. ISS ≥ 25).

Fatal anatomic patterns of injuries were distinguished from those of survivors in two primary ways. First, fatal patterns of injuries were more complex in nature with more numerous and stronger between anatomic region associations that clustered together. Compared to survivors, fatal lower extremity critical injuries (AIS severity 5) from explosive mechanisms were unique as they were positively associated with serious, severe, and critical injuries (AIS severity 3–5) to the abdomen and serious and severe injuries (AIS severity 3–4) to the upper extremities. In addition, fatalities from explosives with these injuries to the abdomen also had associated injuries to the thorax (AIS severity 2–6) while survivors of explosive mechanisms did not have any associated thorax injuries. Second, within anatomic body region associations were often stronger and involved more severe injury groups for fatal injuries. This was especially clear for injuries sustained from a firearm to the thorax and head. Four of the top 10 positive associations for fatal injuries from a firearm involved the following: head severity 6 and 4, head severity 5 and 4, thorax severity 6 and 5, and thorax severity 4 and 3. For survivor injuries from a firearm, these associations were either non-existent or meaningfully weaker.

Based on our study, we propose a three-tiered process for future investigation of anatomic patterns of injury in critically injured casualties. First, using network models, identify meaningful groups of anatomic injuries that appear to distinguish fatal from survivor injuries by injury mechanism. Second, explore specific injuries within these groups to improve discrimination of fatal and survivor anatomic patterns of injuries. Third, link medical interventions and opportunities for improvement to these specific anatomic patterns of injuries. This three-tiered process can lead to insights that will enhance trauma care and trauma systems, mitigate mortality from survivable and potentially survivable injuries, and ultimately reduce preventable deaths.

For example, dismounted complex blast injuries involving traumatic amputations to lower extremities in combination with pelvic, abdominal, and genitourinary injuries are one particular injury pattern previously described as a hallmark of recent conflicts in Afghanistan and Iraq^19–21^. Fatalities can be distinguished from survivors of complex blast injuries involving lower extremities by exploring a similar injury pattern. First, by examining the explosive network models, a total of 240 of 407 (59%) fatalities sustained critical injuries (AIS severity 5) to the lower extremities in combination with severe or critical injuries (AIS severities 4 or 5) of the abdomen, compared to 28 of 142 (20%) of survivors. Given that injuries to the thorax are also positively associated with explosive injuries to the abdomen, inclusion of serious, severe, or critical injuries (AIS severities 3–5) of the thorax to this criteria distinguished fatal [193/407 (47%)] vs. survivor injuries [7/142 (5%)] even further.

Once meaningful anatomic patterns of injury are identified, deeper investigation into specific injuries within these patterns can be explored to distinguish fatal from survivor injury patterns. For example, among casualties of an explosive mechanism who sustained serious injuries (AIS severity 3) as the highest injury severity score for their lower extremities, there is no evidence of survival from an injury pattern consisting of the following three injuries: (1) unilateral below the knee amputation (AIS code: 811003.3), (2) liver laceration greater than 3 centimeters parenchymal depth (AIS code: 541824.3) and (3) hemothorax not further specified (AIS code: 442200.3). This does not necessarily suggest that this specific injury pattern is non-survivable, but possibly a specific meaningful pattern consistently part of a larger more complex pattern that has no documented evidence of survival. This notion is supported by the fact that among the nine fatalities that sustained this injury pattern from an explosive mechanism, all nine also sustained at least one additional severe, critical, or maximum injury (AIS severities 4–6) with an ISS range of 41 to 75. This example highlights how this three-tiered approach can be used to identify more complex injury patterns that consistently have no documented evidence of survival from injuries excluding maximum severity (AIS severity 6) often considered non-survivable by definition.

Using this three-tiered approach we also identified one or more of the following injuries that were common in fatalities (n = 440, 45%) but less common for survivors (n = 59, 20%) of firearm mechanism: (1) complex basilar skull fracture (AIS code: 150206.4); (2) complex vault fracture (AIS code: 150406.4); and/or (3) penetrating injury of the cerebrum greater than 2 cm deep (AIS code: 140692.5). Specific injuries within patterns also highlight variability of injury severity described by one specific code. Particularly problematic are codes for the head. For example, a penetrating injury to the cerebrum of >2 cm includes injuries of 3 cm penetration along with injuries of 15 cm. The same is true for basilar skull fracture which can be linear or complex. The degree of survivability may be found in these inherent coding differences.

Linking specific anatomic patterns of injury with medical interventions and opportunities for improvement can impact efforts toward survivability. For example, despite justifiable focus on hemorrhage as a leading mechanism of death^11–14,22–24^, these results support the need for interventions that not only address the rapid treatment of hemorrhage-related injuries (e.g. traumatic lower extremity amputation and pelvic fractures) but hemorrhage-related injuries in conjunction with injuries that impact respiration (e.g. major lung lacerations to one or both lobes, severe rib fractures, and hemothoraces). The potential clinical importance for future patient care resides with how subject matter experts review injuries for individual fatalities retrospectively in comparison to survivors. A more precise description of traumatic injuries during mortality reviews will facilitate valid and reliable identification of medical and non-medical opportunities for improvement, and subsequently guide priorities for personnel, training, and equipment initiatives to include diagnostics, triage (to optimize resource utilization), therapeutics, and future innovations. This also includes the potential to further inform trauma-associated cardiopulmonary resuscitation guidelines for prehospital providers^25,26^. For example, by identifying and widely distributing circumstances for which specific groups of potentially survivable injuries can often occur together, providers who encounter one of the injuries can also consider the associated injuries and whole-body pattern. Therefore, placing the constellation of injuries in context for optimizing resuscitative care that would ultimately improve survival.

Limitations of our study include the large number of individual and often unique anatomic injuries within each body region for which it was not feasible to explore a network. This reduction of heterogeneity by grouping individual injuries into severity groups by anatomic region results in a loss of information and potentially meaningful differences between individual injuries in the same anatomic severity group. There was also a potential for bias when comparing various networks due to the difference in node number and sample size for these networks^27^. Differential reliability and validity of AIS coding between fatalities and survivors is likely because fatal codes were derived from detailed autopsies while survivor codes were derived from health care records. Although every effort was taken by study investigators to ensure the completeness of records retrospectively, personnel conducting the documentation may have disregarded or underreported anatomic injuries prospectively. It is also possible that other anatomic and/or physiologic injury severity scoring systems not discussed in this study could lead to a different or more nuanced understanding of associations found in these analyses; thus, an area for potential future study. Lastly, this analysis does not consider differences between medical and non-medical factors (e.g. enemy force, environment, logistics) that may have influenced survivability within our study population.

## Conclusion

Understanding injuries, anatomic patterns of injury, and their impact on survivability is complex. The methods used in this study have helped to elucidate injury variability between body regions, mechanisms, and fatalities and survivors. The three-tiered approach presented in this study is unique and has potential to refine objective measures for injury survivability. Given the nearly enumerable combinations of injuries, this approach could prove especially useful for mortality reviews where the objective is not simply to optimize prediction of mortality but also to identify opportunities for improvement specific to each patient’s unique injury pattern. An intimate understanding of injury and injury patterns will enable trauma care and trauma systems to improve survivability.

## Methods

Evaluated were US military casualties, fatalities and survivors, who sustained battle-related critical injuries (ISS 25–75) from October 2001 to December 2014. Because approximately 90% of all deaths occur in casualties with critical injuries (ISS 25–75)^28^, casualties with mild, moderate, or severe injuries (ISS 1–9, 10–15, 16–24, respectively) were excluded from these analyses. Casualties resulting from non-battle manner of injury (e.g. accident, suicide) were also excluded by study definition. The rationale for this exclusion is related to data capture and availability. The Armed Forces Medical Examiner System (AFMES) has medicolegal jurisdiction to complete a forensic pathology investigation, including autopsy, on individuals that die on exclusive federal jurisdiction within the United States and U.S. Service Members that die during combat operations. However, AFMES does not have primary medicolegal jurisdiction on deaths that occur outside of military installations. Additionally, the Department of Defense Trauma Registry (DoDTR) currently has nominal and incomplete capture of data on non-fatal training accidents or suicide attempts for survivor comparison.

Demographic and injury data were obtained from AFMES for fatalities of critical injuries (ISS 25–75), both those who were either Killed in Action (KIA; died before reaching a facility with surgical capability) or Died of Wounds (DOW; died after reaching a facility with surgical capability)^29^. Autopsies were conducted by AFMES forensic pathologists in accordance with National Association of Medical Examiners standards^30^. The documented anatomic injuries were coded by certified coders using the Abbreviated Injury Scale (AIS)-version 2005^31^. Demographic and injury data were obtained from the Joint Trauma System (JTS) DoDTR for survivors of critical injuries (ISS 25–75). The AIS codes for survivors were abstracted from available medical records into the DoDTR by trained and certified medical abstractors from the JTS.

Stratified descriptive comparisons of demographic and injury characteristics were performed for fatalities versus survivors. To perform binary network analyses, AIS codes were categorized into eight primary body regions: head, face, neck, spine, upper extremity, thorax, abdomen, and lower extremity for each possible injury severity (i.e. AIS code post-dot severity of 1–6). Due to their unique nature and circumstance, AIS codes for “other” injury with a severity of 5 or 6 (e.g. AIS code: 060006.5; drowning with cardiac arrest) and “external” injury with a 5 or 6 (e.g. AIS code: 912030.5; 2nd or 3rd degree partial or full-thickness burn of 40–89% total body surface area) were excluded from analysis. Less severe “other” and “external” injuries were rare and not included in the network model analysis. No AIS codes exist for face injuries of severity 5 and 6, upper extremity injuries of 6, or lower extremity injuries of 6; therefore, there were a total of 44 possible anatomical injury combinations. For inclusion in the final stratified analyses, an anatomical injury category required at least 10 fatalities or survivors and could not be an isolated node in the network model.

As the constellation of injuries sustained from trauma are often quite complicated, and the structure of such injuries are not fully understood or based on a known framework, we conceptualized exploring the network of anatomic injury groups for fatalities and survivors similar to the exploration of symptoms of mental health diagnoses in psychopathology^32^. Because sustaining a specific injury is a binary event (yes vs. no) we performed an eLasso binary network analysis^7^ based on an Ising model^33^ using Bayesian neighborhood selection^34–36^ to explore anatomic patterns of injury. These anatomic patterns of injury were explored for both fatalities and survivors for the total population, and for subgroups of casualties injured by explosive and firearm mechanism. Casualties from non-explosive, non-firearm mechanism were insufficient in numbers to perform network models for both fatalities and survivors.

Groups of anatomic injuries by severity were represented as nodes (e.g. head injuries with a severity of 4) and the associations between these groups of injuries were represented by edges. If the association was positive, edges were represented by the color navy blue. If the association was negative, edges were represented by the color orange. The thicker the edge the stronger the positive or negative association. We described the anatomic patterns of injuries within these strata by identifying the structure of anatomic injury patterns and local measures of centrality (i.e. strength, closeness, betweenness). Strength is a measure of the number and strength of connections for a specific anatomical injury group. Closeness measures how close the anatomical injury group of interest is to other anatomical injury groups. Betweenness is a measure of how likely an anatomical group of interest connects or bridges other anatomical groups in the network. We also performed heat maps of the associations using odd ratios within and between anatomic body regions by mechanism and reported the top 10 positive associations. The total number of connections, positive connections, negative connections, average shortest path, smallworldness^9^, and transitivity^37^ were reported as general descriptions of the networks. Given the analysis included the entire population of fatal injuries, and the majority of critically injured survivor injuries^28^, instability in the networks is likely due to the inherent variability of injuries from the population rather than sampling bias. Therefore, network stability metrics were not performed.

This performance improvement project was approved as not human subjects research by the US Army Institute of Surgical Research as defined in 32 CFR 219.102(e) and in accordance with relevant guidelines and regulations as implemented through DoDI 3216.02^38^. As such, the documentation of informed consent does not apply for not human subjects research. The binary network models were performed using the IsingFit package^7^, visualized with the qgraph^39^ package, and heatmaps were produced using the gplots package^40^ in R (version 3.5.1). All other analyses were performed in SAS (Cary, NC) version 9.4.

## Supplementary information

Supplementary Material

## Data Availability

The data that support the findings of this study are available from the Defense Health Agency but restrictions apply to the availability of these data, which were used as part of a US Department of Defense regulatory approved performance improvement project, and so are not publicly available.
