# Supplementary material for: Patterns of Anatomic Injury in Critically Injured Combat Casualties: A Network Analysis
Source: Sci Rep. 2019 Sep 24;9:13767. doi: 10.1038/s41598-019-50272-3 (PMC6760527; doi:10.1038/s41598-019-50272-3)
Supplement: Supplementary file 1 — Supplementary Material [file 41598_2019_50272_MOESM1_ESM.docx]

**Patterns of Anatomic Injury in Critically Injured Combat Casualties: A Network Analysis**

Jud C. Janak, PhD; Edward L. Mazuchowski; MD, PhD; Russ S. Kotwal, MD, MPH; Zsolt T. Stockinger, MD; Jeffrey T. Howard, PhD; Frank K. Butler, MD; Jonathan A. Sosnov, MD; Jennifer M. Gurney, MD; Stacy A. Shackelford, MD

**eTable 1.** Number of Connections Within and Between Anatomic Injury Groups among Fatalities of US Military Casualties who Sustained Battle-Related Critical Injuries (Injury Severity Scale Score, 25-75) from Explosive Mechanism (N=3,037).

|  | **Head** | **Neck** | **Face** | **Spine** | **Thorax** | **Upper Extremity** | **Abdomen** | **Lower Extremity** |
| --- | --- | --- | --- | --- | --- | --- | --- | --- |
| Head | **10/15 (67)** |  |  |  |  |  |  |  |
| Neck | 4/36 (11) | **7/15 (47)** |  |  |  |  |  |  |
| Face | 10/18 (56) | 6/18 (33) | **1/3 (33)** |  |  |  |  |  |
| Spine | 3/24 (13) | 4/24 (17) | 2/12 (17) | **2/6 (33)** |  |  |  |  |
| Thorax | 5/36 (14) | 1/36 (3) | 0/18 (0) | 5/24 (21) | **10/15 (67)** |  |  |  |
| Upper Extremity | 3/30 (10) | 4/30 (13) | 4/15 (27) | 2/20 (10) | 5/30 (17) | **2/10 (20)** |  |  |
| Abdomen | 5/36 (14) | 1/36 (3) | 2/18 (11) | 4/24 (17) | 19/36 (53) | 5/30 (17) | **10/15 (67)** |  |
| Lower Extremity | 4/30 (13) | 1/30 (3) | 1/15 (7) | 1/20 (5) | 4/30 (13) | 6/25 (24) | 13/30 (43) | **3/10 (30)** |
| ***All other Anatomic Regions*** | ***34/210 (16)*** | ***21/210 (10)*** | ***25/114 (22)*** | ***23/148 (16)*** | ***39/210 (19)*** | ***29/180 (16)*** | ***49/210 (23)*** | ***30/180 (17)*** |

Number of connections within and between Abbreviated Injury Scale anatomic injury groups relative to the maximum number of possible connections. Within anatomic region associations are in bold.

**eTable 2.** Number of Connections Within and Between Anatomic Injury Groups among Survivors of US Military Casualties who Sustained Battle-Related Critical Injuries (Injury Severity Scale Score, 25-75) from Explosive Mechanism (N=1,069).

|  | **Head** | **Neck** | **Face** | **Spine** | **Thorax** | **Upper Extremity** | **Abdomen** | **Lower Extremity** |
| --- | --- | --- | --- | --- | --- | --- | --- | --- |
| Head | **5/10 (50)** |  |  |  |  |  |  |  |
| Neck | 0/20 (0) | **5/6 (83)** |  |  |  |  |  |  |
| Face | 4/15 (27) | 1/12 (8) | **2/3 (67)** |  |  |  |  |  |
| Spine | 0/25 (0) | 0/20 (0) | 0/15 (0) | **5/10 (50)** |  |  |  |  |
| Thorax | 1/20 (5) | 0/16 (0) | 0/12 (0) | 1/20 (5) | **5/6 (83)** |  |  |  |
| Upper Extremity | 0/20 (0) | 0/16 (0) | 2/12 (17) | 0/20 (0) | 1/16 (6) | **3/6 (50)** |  |  |
| Abdomen | 2/25 (8) | 0/20 (0) | 1/15 (7) | 0/25 (0) | 0/20 (0) | 1/20 (5) | **6/10 (60)** |  |
| Lower Extremity | 4/25 (16) | 0/20 (0) | 0/15 (0) | 1/25 (4) | 1/20 (5) | 0/20 (0) | 5/25 (20) | **5/10 (50)** |
| ***All other Anatomic Regions*** | ***11/150 (7)*** | ***1/128 (1)*** | ***8/96 (8)*** | ***2/150 (1)*** | ***4/124 (3)*** | ***4/124 (3)*** | ***9/150 (6)*** | ***11/150 (7)*** |

Number of connections within and between Abbreviated Injury Scale anatomic injury groups relative to the maximum number of possible connections. Within anatomic region associations are in bold.

**eTable 3.** Number of Connections Within and Between Anatomic Injury Groups among Fatalities of US Military Casualties who Sustained Battle-Related Critical Injuries (Injury Severity Score 25-75) from Firearm Mechanism (N=988).

|  | **Head** | **Neck** | **Face** | **Spine** | **Thorax** | **Upper Extremity** | **Abdomen** | **Lower Extremity** |
| --- | --- | --- | --- | --- | --- | --- | --- | --- |
| Head | **9/15 (60)** |  |  |  |  |  |  |  |
| Neck | 0/18 (0) | **2/3 (67)** |  |  |  |  |  |  |
| Face | 1/12 (8) | 0/6 (0) | **1/1 (100)** |  |  |  |  |  |
| Spine | 0/18 (0) | 1/9 (11) | 1/6 (17) | **1/3 (33)** |  |  |  |  |
| Thorax | 3/36 (8) | 0/18 (0) | 0/12 (0) | 1/18 (6) | **8/15 (53)** |  |  |  |
| Upper Extremity | 0/18 (0) | 0/9 (0) | 0/6 (0) | 0/9 (0) | 2/18 (11) | **1/3 (33)** |  |  |
| Abdomen | 0/30 (0) | 0/15 (0) | 0/10 (0) | 0/15 (0) | 4/30 (13) | 1/15 (7) | **6/10 (60)** |  |
| Lower Extremity | 0/24 (0) | 0/12 (0) | 0/8 (0) | 0/12 (0) | 0/24 (0) | 3/12 (25) | 5/20 (25) | **2/6 (33)** |
| ***All other Anatomic Regions*** | ***4/156***  ***(3)*** | ***2/87***  ***(2)*** | ***2/60***  ***(3)*** | ***3/87***  ***(3)*** | ***10/156***  ***(6)*** | ***6/87***  ***(7)*** | ***10/135***  ***(7)*** | ***8/135***  ***(6)*** |

Number of connections within and between Abbreviated Injury Scale anatomic injury groups relative to the maximum number of possible connections. Within anatomic region associations are in bold.

**eTable 4.** Number of Connections Within and Between Anatomic Injury Groups among Survivors of US Military Casualties who Sustained Battle-Related Critical Injuries (Injury Severity Scale Score, 25-75) from Firearm Mechanism (N=301).

|  | **Head** | **Neck** | **Face** | **Spine** | **Thorax** | **Upper Extremity** | **Abdomen** | **Lower Extremity** |
| --- | --- | --- | --- | --- | --- | --- | --- | --- |
| Head | **6/6 (100)** |  |  |  |  |  |  |  |
| Neck | 0/16 (0) | **3/6 (50)** |  |  |  |  |  |  |
| Face | 1/8 (13) | 0/8 (0) | **1/1 (100)** |  |  |  |  |  |
| Spine | 0/12 (0) | 0/12 (0) | 0/6 (0) | **2/3 (67)** |  |  |  |  |
| Thorax | 0/16 (0) | 0/16 (0) | 0/8 (0) | 0/12 (0) | **3/6 (50)** |  |  |  |
| Upper Extremity | 0/12 (0) | 0/12 (0) | 0/6 (0) | 0/9 (0) | 0/12 (0) | **2/3 (67)** |  |  |
| Abdomen | 1/16 (6) | 1/16 (6) | 0/8 (0) | 0/12 (0) | 0/16 (0) | 0/12 (0) | **4/6 (67)** |  |
| Lower Extremity | 0/16 (0) | 0/16 (0) | 0/8 (0) | 0/12 (0) | 0/16 (0) | 0/12 (0) | 2/16 (13) | **3/6 (50)** |
| ***All other Anatomic Regions*** | ***2/96 (2)*** | ***2/96 (2)*** | ***1/52 (2)*** | ***0/75 (0)*** | ***0/96 (0)*** | ***0/75 (0)*** | ***4/96 (4)*** | ***2/96 (2)*** |

Number of connections within and between Abbreviated Injury Scale anatomic injury groups relative to the maximum number of possible connections. Within anatomic region associations are in bold.

**eTable 5**. Top 10 Positive Associations Within and Between Anatomic Body Region with Abbreviated Injury Scale (AIS) Score of Severity among Fatalities of US Military Casualties who Sustained Battle-Related Critical Injuries (Injury Severity Scale Score, 25-75), Stratified by Injury Mechanism.

| **Mechanism** | **Anatomic Region 1 (AIS Severity)** | **Anatomic Region 2 (AIS Severity)** | **Odds Ratio** |
| --- | --- | --- | --- |
| ***Overall*** | 1. Neck (5) | Neck (4) | 19.0 |
|  | 1. Head (5) | Head (4) | 17.4 |
|  | 1. Thorax (6) | Thorax (5) | 13.2 |
|  | 1. Neck (4) | Neck (3) | 9.9 |
|  | 1. Head (6) | Head (4) | 8.9 |
|  | 1. Neck (3) | Neck (2) | 7.3 |
|  | 1. Head (4) | Face (3) | 6.3 |
|  | 1. Lower Extremity (3) | Lower Extremity (2) | 6.2 |
|  | 1. Neck (6) | Upper Extremity (4) | 5.9 |
|  | 1. Upper Extremity (1) | Lower Extremity (1) | 4.6 |
| ***Explosive*** | 1. Head (5) | Head (4) | 11.8 |
|  | 1. Neck (5) | Neck (4) | 11.5 |
|  | 1. Thorax (6) | Thorax (5) | 11.2 |
|  | 1. Neck (4) | Neck (3) | 7.3 |
|  | 1. Neck (3) | Neck (2) | 7.1 |
|  | 1. Head (6) | Head (4) | 7.0 |
|  | 1. Head (5) | Head (2) | 6.2 |
|  | 1. Head (3) | Head (2) | 5.9 |
|  | 1. Head (4) | Face (3) | 5.9 |
|  | 1. Lower Extremity (3) | Lower Extremity (2) | 5.5 |
| ***Firearm*** | 1. Head (5) | Head (4) | 80.2 |
|  | 1. Head (6) | Head (4) | 75.6 |
|  | 1. Neck (4) | Neck (3) | 12.6 |
|  | 1. Thorax (6) | Thorax (5) | 9.3 |
|  | 1. Neck (3) | Neck (2) | 6.7 |
|  | 1. Lower Extremity (3) | Lower Extremity (2) | 5.8 |
|  | 1. Thorax (4) | Thorax (3) | 4.5 |
|  | 1. Abdomen (4) | Abdomen (3) | 4.5 |
|  | 1. Head (4) | Head (1) | 4.3 |
|  | 1. Head (3) | Head (2) | 3.7 |

AIS Severity-1: minor, 2: moderate, 3: serious, 4: severe, 5: critical, 6: maximum

**eTable 6.** Top 10 Positive Associations Within and Between Anatomic Body Region with Abbreviated Injury Scale (AIS) Score of Severity among Survivors of US Military Casualties who Sustained Battle-Related Critical Injuries (Injury Severity Scale Score, 25-75), Stratified by Injury Mechanism.

| **Mechanism** | **Anatomic Region 1 (AIS Severity)** | **Anatomic Region 2 (AIS Severity)** | **Odds Ratio** |
| --- | --- | --- | --- |
| ***Overall*** | 1. Face (4) | Face (3) | 14.5 |
|  | 1. Neck (4) | Neck (3) | 12.8 |
|  | 1. Spine (3) | Spine (2) | 10.4 |
|  | 1. Face (2) | Face (1) | 9.2 |
|  | 1. Lower Extremity (3) | Lower Extremity (2) | 8.5 |
|  | 1. Thorax (3) | Thorax (2) | 7.4 |
|  | 1. Upper Extremity (4) | Upper Extremity (3) | 7.0 |
|  | 1. Head (4) | Head Severity (3) | 6.9 |
|  | 1. Upper Extremity (3) | Upper Extremity (2) | 6.8 |
|  | 1. Spine (5) | Spine (2) | 6.1 |
| ***Explosive*** | 1. Neck (4) | Neck (3) | 11.7 |
|  | 1. Spine (3) | Spine (2) | 9.8 |
|  | 1. Face (2) | Face (1) | 7.4 |
|  | 1. Lower Extremity (3) | Lower Extremity (2) | 7.2 |
|  | 1. Head (4) | Head (3) | 6.5 |
|  | 1. Upper Extremity (4) | Upper Extremity (3) | 6.4 |
|  | 1. Neck (5) | Neck (3) | 6.0 |
|  | 1. Abdomen (3) | Abdomen (2) | 5.8 |
|  | 1. Upper Extremity (3) | Upper Extremity (2) | 5.7 |
|  | 1. Thorax (3) | Thorax (2) | 5.5 |
| ***Firearm*** | 1. Head (5) | Head (3) | 25.3 |
|  | 1. Lower Extremity (3) | Lower Extremity (2) | 15.1 |
|  | 1. Thorax (3) | Thorax (2) | 14.6 |
|  | 1. Face (2) | Face (1) | 12.4 |
|  | 1. Head (4) | Head (3) | 10.7 |
|  | 1. Upper Extremity (3) | Upper Extremity (2) | 8.9 |
|  | 1. Neck (4) | Neck (3) | 8.7 |
|  | 1. Neck (4) | Neck (2) | 7.5 |
|  | 1. Lower Extremity (4) | Lower Extremity (3) | 7.4 |
|  | 1. Neck (3) | Neck (2) | 5.8 |

AIS Severity-1: minor, 2: moderate, 3: serious, 4: severe, 5: critical, 6: maximum

**eFigure 1.** Flow diagram of final analytic sample

**Critically Injured Fatalities**

28

No AIS Codes

**Critically Injured Survivors**

79

External/Other Trauma Severity ≥5

16,464

ISS 1-24

253

Injured after 2014

261

Deceased

51,360

Non-US Military

103

Deceased in AFMES

13,936*

Non-Battle Injured

83,839 Injured

32,479 Injured

18,543 Battle Injured

18,282 Battle Injured

18,029 Battle Injured

*10 patients excluded with unknown status

1,383 Battle Injured

1,462 Battle Injured

17,926 Battle Injured

5,344 Battle Deaths

450

External/Other Trauma Severity ≥5

4,770 Battle Deaths

4,320 Battle Deaths

5,262 Battle Deaths

492

ISS 1-24

54

Complex Mechanism

5,316 Battle Deaths

**eFigure 2.** Network Structure Local Measures of Centrality among Fatal and Survivor Battle Injured US Service Members, Stratified by Injury Mechanism

**
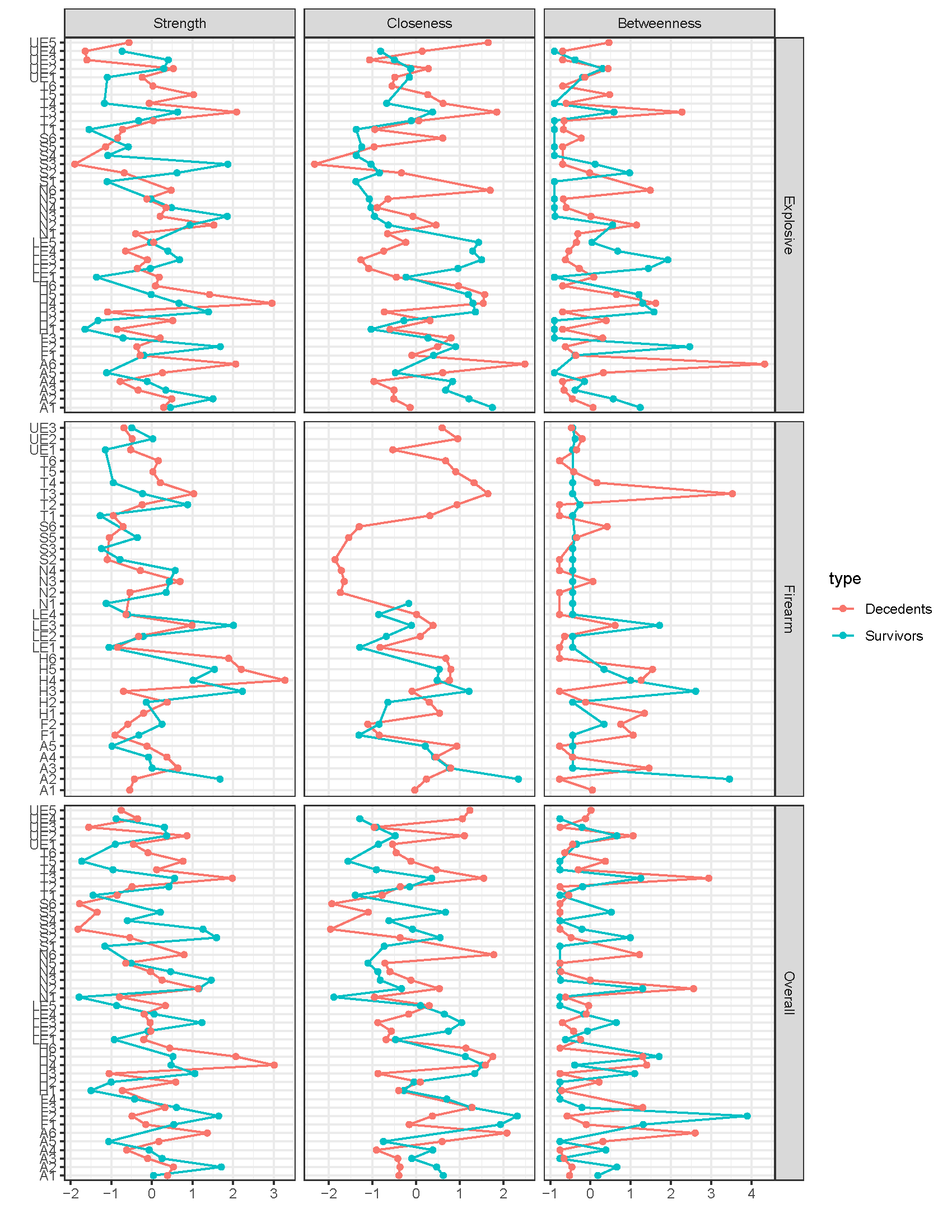
**

Individual injuries grouped by both the anatomic body region injured (H: Head, F: Face, N: Neck, T: Thorax, A: Abdomen, S: Spine, UE: Upper Extremity, LE: Lower Extremity) and severity of the injury (1: minor, 2: moderate, 3: serious, 4: severe, 5: critical, 6: maximum). For example, A3 is equivalent to all Abbreviated Injury Scale-2005 Injuries to the abdomen with a severity of 3 or serious.
